# Influence of resilience on the relations among acculturative stress, somatization, and anxiety in latinx immigrants

**DOI:** 10.1002/brb3.1863

**Published:** 2020-09-29

**Authors:** Annahir N. Cariello, Paul B. Perrin, Alejandra Morlett‐Paredes

**Affiliations:** ^1^ Department of Psychology Virginia Commonwealth University Richmond VA USA; ^2^ Department of Psychiatry University of California San Diego CA USA

**Keywords:** anxiety, Latin immigration, resilience, somatization

## Abstract

**Objective:**

In cultures where psychological distress is stigmatized, the presentation of emotional distress as somatic complaints is a frequent occurrence. Understanding factors that contribute to the presentation of somatization in Latinx immigrants is crucial due to its tie to poor quality of life. The purpose of this study was to explore relations among acculturative stress, anxiety, somatization, and resilience in a sample of Latinx immigrants living in the United States.

**Methods:**

Data were collected from 204 Latinx immigrants across diverse community settings.

**Results:**

Acculturative stress was positively related to both anxiety and somatization, and the relation between acculturative stress and somatization occurred through anxiety. Resilience moderated the relations between acculturative stress and somatization, and between anxiety and somatization.

**Conclusion:**

This study suggests that Latinx immigrants presenting with somatic symptoms may benefit from the examination of a possible comorbid presentation of anxiety or acculturative stressors. An integrated behavioral healthcare approach is recommended when working with Latinx immigrants evaluating the impact of minority stressors on health. Clinicians are encouraged to incorporate cultural protective factors that reinforce the development and sustenance of resilience.

## Graphical abstract

The purpose of this study was to explore relations among acculturative stress, anxiety, somatization, and resilience in a sample of Latinx immigrants living in the United States. Acculturative stress was positively related to both anxiety and somatization, and the relation between acculturative stress and somatization occurred through anxiety. Resilience moderated the relations between acculturative stress and somatization, and between anxiety and somatization.

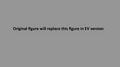

## 1 INTRODUCTION

In many cultures, psychosocial stressors present clinically as somatization (Shiroma & Alarcon, 2011) “the presentation of one or more medically unexplained somatic symptoms related to substantial emotional distress” (Escobar et al., 1987, p. 713). In primary care, approximately two‐thirds of symptoms are medically unexplained, resulting in estimates of 16%–34% of patients with possible somatization (Haller et al., 2015; Steinbrecher et al., 2011). Not only is somatization common, but it is often unrecognized, severely underdiagnosed, disabling, and associated with notable impairment (Dimsdale et al., 2013; Hamilton et al., 2013; Levenson, 2011). Among individuals of diverse ethnic and cultural backgrounds, somatization is one of the leading causes of under‐recognition and undertreatment of mental health concerns (Ferrari et al., 2015). To understand the presentation of somatic complaints, examination of interactions among physiological, psychological, social, and cultural factors is required (Creed et al., 2012; Ferrari et al., 2015; Tanaka et al., 2011). Psychological factors are associated with severity of somatic symptoms (Creed et al., 2011; Hyphantis et al., 2013). Evidence is growing supporting somatization not only has cultural, social, psychological, and physical explanations, but also several of these explanations operating simultaneously (Frostholm et al., 2010; Martin & Rief, 2011; Rief et al., 2004). In cultures where psychological distress is stigmatized, including Latinx immigrants, the presentation of emotional distress as somatic complaints is a frequent occurrence (Kleinman, 1982). As Latinx immigrants experience substantially more stressful and traumatic events than nonimmigrants (Rousseau & Drapeau, 2004), resulting in higher levels of psychological distress, this group may be more likely to experience a somatization of psychosocial issues compared with nonimmigrant populations (Ritsner et al., 2000). As such, the purpose of this study was to investigate the relations among anxiety, somatization, acculturative stress, and resilience in Latinx immigrants living in the United States.

### 1.1 Minority stress model

Minority stress is experienced by individuals in minority positions from stigmatized social categories, racial and ethnic minorities, including Latinx immigrants (Meyer, 2003). Health disparities found within these racial and ethnic groups may be attributed to inequality expressed in social and economic disadvantages (Spalter‐Roth et al., 2005). Individuals in minority positions experience multiple adverse conditions including poverty, discrimination, inadequate healthcare services, and/or housing which negatively impact both mental and physical health (Williams et al., 1997). In addition to minority stressors, racial and ethnic minorities experience daily life stressors heightening potential health risks (Turner & Avison, 2003). Meyer's (2003) minority stress model describes the unique stressors experienced by minority statuses, its impact on mental and physical health, and strengths that buffer its effects. Empirical support for the negative impact of minority stressors, including acculturative stress, on the mental and physical health of Latinxs is growing (Torres & Wallace, 2013). Congruent with Meyer's (2003) minority stress model, we hypothesize acculturative stress in Latinx immigrants negatively impacts their physical health expressed in somatization, and this relationship will be explained through increased levels of anxiety. The minority stress model also emphasizes protective cultural factors that weaken these relationships (Meyer, 2003), and we hypothesize resilience may be a primary protective mechanism in Latinx immigrants.

### 1.2 Somatization and Latinx

In some Latinx countries, somatic symptoms are more closely linked to psychosocial distress than a diagnosed medical condition, suggesting cultural patterning of somatic symptom presentation wherein the clinical presentation of physical symptoms is embedded in culture (Escobar & Gureje, 2007; Sumathipala et al., 2008). Latinxs report substantially more somatic symptoms compared with non‐Latinx Whites in the United States (Escobar et al., 1983). Escobar and Canino (1989) found greater frequency of somatic complaints among Latinx immigrants (2.6 functional symptoms) than Latinx Americans (1.4 functional symptoms). In a sample of Puerto Ricans living in the United States, psychological distress was found to be closely connected to pain, suggesting Latinx immigrants may be more expressive of somatic complaints when distressed (Lipton & Marbach, 1984). As somatic symptoms are the leading cause of outpatient medical visits and worsened quality of life in the United States, investigation of factors that contribute to somatization in Latinx immigrants living in the United States is critical (Dimsdale & Creed, 2009; Kroenke, 2003; Rief et al., 2010).

### 1.3 Somatization and anxiety

Extensive evidence supports the association between anxiety disorders and somatic symptoms in U.S. samples (Egger et al., 1999; van Boven et al., 2011) wherein somatic symptoms worsen with distressing and impairing thoughts, behaviors, and feelings (Dimsdale et al., 2013). Creed et al. (2012) found that anxiety symptoms were associated with physical discomfort. In population‐ based cohort studies persistent and multiple somatic complaints have been associated with anxiety and depression (Haug et al., 2004; Leiknes et al., 2007). The presentation of somatic symptoms is tied to a twofold increased risk of an anxiety disorder (Kroenke et al., 1997). Among Latinx individuals living in the United States, somatic symptoms are associated with mental health issues including depression, anxiety, and substance use disorders (Escobar et al., 2010). Latinx immigrant adolescents have a more severe presentation of anxiety and somatic complaints compared with Spaniard immigrant adolescents living in the United States, and the authors attributed the finding to possible cultural differences of individualism as compared to collectivism (Romero‐Acosta et al., 2014). Mexican and Puerto Rican immigrants living in California who met criteria for anxiety disorders, major depression, dysthymia, and schizophrenia also reported high frequencies of somatic complaints (Canino, 1988). Comorbid somatic complaints and anxiety are associated with greater medical care usage than each of these conditions alone (Barsky et al., 2005; De Waal et al., 2004). Bauer et al. (2012) found Latinx immigrants with somatic complaints were more likely to self‐disclose the need for mental health services compared with second‐generation Latinxs. In addition, providers are less likely to talk about mental disorders with racial and ethnic minority patients compared with White patients, resulting in under‐recognition of these concerns and lack of referrals to mental health providers (Borowsky et al., 2000; Talbott, 2009). To the best of our knowledge, no research to date has investigated the association between anxiety and somatization in Latinx adult immigrants living in the United States. As such, a greater understanding of the associations between anxiety and somatic complaints, and the potential factors that impact this relationship in Latinx immigrants is crucial to decrease possible health care disparities.

### 1.4 Latinx acculturative stress

Minority stressors have been linked to psychological distress (Moritsugu & Sue, 1983; Smedley et al., 1993), as well as acculturative stress, and in particular has been found to negatively impact Latinx mental and physical health (Finch & Vega, 2003; Wong et al., 2017). Acculturation is the dual process of cultural and psychological change as a result of contact between two or more cultural groups, and acculturative stress is the loss experienced during this adjustment (Hovey, 2000). Alamilla et al. (2010) found an association between minority stressors and somatization. As more acculturated individuals experience increased awareness of and exposure to discrimination, increased family cultural conflict, and detrimental effects of assimilation of unhealthy behaviors report poorer mental and physical health, it is possible that acculturative stress impacts somatic symptoms (Bauer et al., 2012). Shiroma and Alarcon (2011) found length of stay in the United States to be positively associated with somatization in Latinx immigrants. In addition, Mexican Americans with low or medium levels of acculturation reported more somatic complaints (Escobar et al., 1987). Given that 80% of Latinx immigrants whose countries of origin span Mexico and Central America report experiencing acculturative stress, it is important to examine its impact on anxiety and somatization (Arbona et al., 2010). Further, it is possible that acculturative stress affects somatization via anxiety. Although this mediation has not been previously investigated, prior work in Latinx immigrants has supported relations between (a) acculturative stress and anxiety, and (b) anxiety and somatization. No investigation to date has evaluated the possible relation between acculturative stress and somatization in Latinx immigrants.

### 1.5 Latinx resilience

Empirical support has been found for associations among somatization, anxiety, and acculturative stress, and these variables have also been linked to resilience (Abraído‐Lanza et al., 2004; Arredondo et al., 2005; Finch et al., 2000; Mulvaney‐Day et al., 2007; Torres, 2009). Resilience is defined as “a dynamic process encompassing positive adaptation within the context of significant adversity” (Luthar & Cicchetti, 2000, p. 1). Positive adaptation occurs when an individual expresses behavioral, social, or interpersonal competence (Luthar & Cicchetti, 2000; Luthar et al., 2000). In Latinx immigrants, resilience is often expressed as cultural protective factors including familism, religiosity, biculturalism, personalism, and community support (Luthar et al., 2000). Torres (2009) found resilience mediated the relationship between mental health concerns and minority stressors in Latinx college students. In addition, resilience was linked with lower mental health symptoms and greater reported health in Latinx immigrants (Abraído‐Lanza et al., 2004; Arredondo et al., 2005; Finch et al., 2000; Mulvaney‐Day et al., 2007). Therefore, there is substantial evidence linking resilience with advantageous mental and physical health outcomes. Latinxs’ utilization of cultural strengths expressed as resilience may possibly be acting as a buffer to minority stressors.

### 1.6 Current study

**Figure 1:**
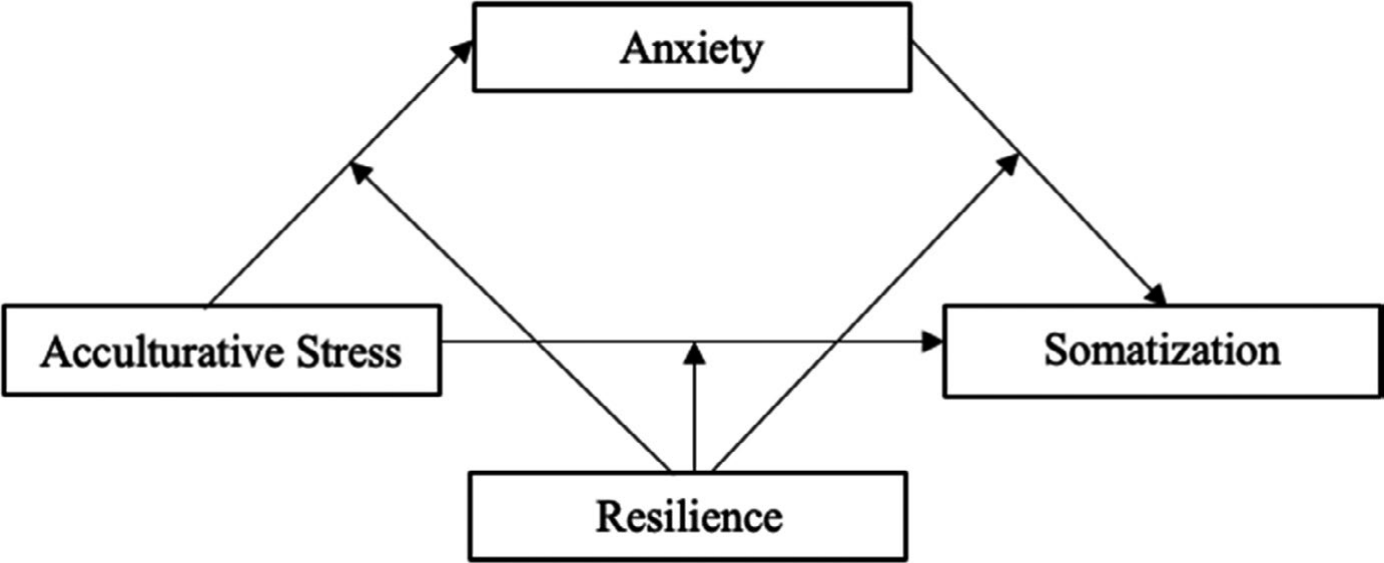
Relationships among acculturative stress, anxiety, and somatization and these direct and indirect effects moderated by resilience. The c path represents the total effect of acculturative stress on somatization. The c′ path represents the effect of acculturative stress on somatization after controlling for anxiety

Research on the impact of acculturative stress on somatization in Latinx immigrants is extremely limited. Additional investigations of the prevalence and severity of somatization in Latinx immigrants are also needed, and the impact of anxiety on Latinx immigrants’ somatization is not well understood. Thus, investigation of the impact of acculturative stress on Latinx immigrant anxiety and somatization is warranted. As resilience has been found to positively impact Latinx mental and physical health, investigation of its possible buffering effects on these relationships is necessary to understand and implement culturally sensitive interventions to counteract the negative effects of this minority stressor. Although studies have examined associations among acculturative stress, anxiety, somatization, and resilience, no study has explored interconnections and pathways that may link these constructs in Latinx immigrants. The primary aim of the current study was to examine the relations among acculturative stress, anxiety, and somatization in a sample of Latinx immigrants living in the United States. A secondary aim was to examine whether the direct and indirect effects among these series of variables are moderated by resilience. A visual representation of these relationships and model appears in Figure 1.

## 2 METHOD

### 2.1 Participants

Participants were recruited as part of a convenience sample from the general Richmond, Virginia city area and surrounding counties. An initial community sample of 207 participants was recruited from churches, restaurants, barber shops, primary care clinics, social service organizations, and Latinx sports associations, among other similar community organizations. There were a number of inclusion criteria for participants. Participants must have been (a) born in Latin America (including Puerto Rico and Brazil); (b) over the age of 18; and (c) able to read and write in Spanish via self‐report. To ensure participants met these criteria, they were prescreened prior to beginning the informed consent. Of these initial 207 participants, three participants’ data were removed from the database due to greater than 50% missingness. As a result, the final sample size was *N* = 204. The average age of these 204 participants was 36.63 (*SD* = 12.45), most were women (64.2%), married (47.5%), and with a family gross income below $15,000 (42.2%). Participants’ demographics appear in Table 1.

**Table 1:** Patient characteristics

Variable	Participants (*n* = 204)
Age, *M* (*SD*)	36.63 (12.45)
Years in the United States, *M* (*SD*)	14.01 (11.92)
Sex, *n* (%)	
Female	69 (33.8)
Male	131 (64.2)
Nationality, *n* (%)	
Mexican	57 (27.9)
Salvadoran	41 (20.1)
Guatemalan	31 (15.2)
Honduran	19 (9.3)
Dominican	11 (5.4)
Peruvian	7 (3.4)
Colombian	6 (2.9)
Nicaraguan	3 (1.5)
Bolivian	3 (1.5)
Cuban	3 (1.5)
Venezuelan	2 (1.0)
Argentinian	1 (0.5)
Brazilian	1 (0.5)
Paraguayan	1 (0.5)
Other (Hispanic)	15 (8.9)
Family gross income *n* (%)	
>$15,000	86 (8.8)
$15,000–$35,000	52 (25.5)
$35,000–$55,000	28 (13.7)
$55,000–$75,000	11 (5.4)
<$75,000	9 (4.4)
Highest education acquired, *n* (%)	
Primary	45 (22.1)
Secondary	33 (16.2)
High School/GED	45 (22.1)
Some College	34 (16.7)
University	31 (15.2)
Postgraduate	11 (5.4)
Marital status, *n* (%)	
Married	98 (48)
Single	64 (31.4)
Open Union	18 (8.8)
Divorced	5 (2.5)
Separated	5 (2.5)
Widowed	1 (0.5)

### 2.2 Measures

All scales used in the current study had a validated Spanish version by similar Latinx samples in the United States and were readily available.

#### 2.2.1 Patient Health Questionnaire‐15 (PHQ‐15)

The PHQ‐15 is a health questionnaire made up of 15 possible physical problems including stomach pain, headaches, back pain, pain in legs, arms, and joints (Wulsin et al., 2002) evaluating symptoms over the past month. In health care (Löwe et al., 2008; van Ravesteijn et al., 2009) and research (Kocalevent et al., 2013; for an overview see Kroenke et al., 2010), the PHQ‐15 is a screening instrument for somatization syndromes, and for the purposes of this study, it was used to investigate somatization. The PHQ‐15 scores each of the possible physical problems as “0” (“not bothered”) to “2” (“a lot”). Scoring in the current study used the total score ranging from 0 to 30 points; higher scores indicate higher levels of physical problems in daily life. The PHQ‐15 has been found to have good internal reliability with a Cronbach's alpha = 0.87 and adequate test–retest reliability among Latinx samples living in the United States (Wulsin et al., 2002).

#### 2.2.2 Generalized Anxiety Disorder‐7 (GAD‐7)

GAD‐7 is a 7‐item anxiety assessment with item responses including “0” (not at all), “1” (several days), “2” (more than half the days), and “3” (nearly every day) (Spitzer et al., 2006). Scoring used the mean of all items; higher mean scores indicate higher levels of anxiety. The GAD‐7 has been found to have excellent internal reliability with a Cronbach's alpha = 0.92 and good test–retest reliability in Latinx samples living in the United States (Spitzer et al., 2006).

#### 2.2.3 Brief Resilience Scale (BRS)

The BRS assesses the ability to bounce back or recover from stress (Rodríguez‐Rey et al., 2016). Scoring in the current study used the mean of all items; higher scores indicate higher resilience. The BRS has been found to have moderately good internal reliability with a Cronbach's alpha = 0.83 and moderate test–retest reliability among Latinx samples living in the United States (Rodríguez‐Rey et al., 2016).

#### 2.2.4 Riverside Acculturation Stress Inventory (RASI)

The RASI measures 5 domains of acculturative stress including intercultural relations, language skills, discrimination, work challenges, and cultural/ethnic makeup of the community (Benet‐Martinez, 2003). The inventory is comprised of 15 items, each rated on a 5‐point scale ranging from 1 (strongly disagree) to 5 (strongly agree). The total score was used for the current study with higher scores reflecting higher levels of acculturative stress. The RASI has been found to have moderately strong internal reliability with a Cronbach's alpha = 0.85 in Latinx samples living in the United States (Miller et al., 2011).

### 2.3 Procedure

This study was approved by the host university's Institutional Review Board. Participants who met the criteria were provided an informed consent form for the survey which they signed. After consenting, participants completed the questionnaires and demographic information, and they were paid an incentive of $5 cash.

### 2.4 Data Analysis plan

#### 2.4.1 Preliminary analyses

Prior to conducting the primary statistical analyses reflecting the study's aims, descriptive statistics (i.e., means, standard deviations, and ranges) of participants’ acculturative stress, anxiety, somatization, and resilience scales were computed. Based on the clinical cutoff scores empirically derived by scale developers in Latinx samples living in the United States, the percentage of participants who reported clinically significant scores on the anxiety measure were calculated.

Normality tests (i.e., skewness and kurtosis) were conducted to determine whether the scales were normally distributed. Critical values of 2.0 were used to identify variables that were skewed or kurtotic. Data were checked for multicollinearity via correlation coefficients among all independent variables (with a goal *r* < .70 among all predictors). To examine bivariate correlations among acculturative stress, anxiety, somatization, and resilience, a correlation matrix was created. Missing data were imputed using the expectation–maximization algorithm.

One mediational model was developed using the PROCESS macro (Hayes, 2014) such that acculturative stress was specified to lead to anxiety, which was specified to lead to somatization. Subsequently, the mediational model was expanded to a moderated mediation with the PROCESS macro examining resilience as a possible moderated of these direct and indirect effects.

## 3 RESULTS

### 3.1 Preliminary analyses

#### 3.1.1 Normality and multicollinearity

Tests of skewness and kurtosis suggested that most variables were below the 2.0 cutoff in terms of skewness, but one variable was somewhat kurtotic: somatization (2.52). Given the general pattern of normality among the variables and the challenges in interpreting transformed data, this variable was retained in its original form. Multicollinearity was checked via correlation coefficients among all primary study variables (value *r* < .70), and no variables were correlated above this threshold.

#### 3.1.2 Descriptive statistics

Descriptive statistics for the measures of acculturative stress, anxiety, somatization, and resilience appear in Table 2, as well as correlations among all primary study variables. Based on the clinical cutoff of 5 for GAD‐7 validated in Latinx samples in the United States (Spitzer et al., 2006), 34.9% of participants met or surpassed the threshold for clinically significant anxiety symptoms, and 18.6%, 9.7%, and 6.6% endorsed mild, moderate, and severe symptoms, respectively. For the PHQ‐15, scores of 5, 10, and 15 represent cutoff points for low, medium, and high (Kocalevent et al., 2013), and 58.3%, 23.1%, and 18.6% of participants endorsed each level of somatic symptom severity, respectively.

**Table 2:** Overall correlation matrix

	1	2	3	4	*M*	*SD*	Range
1. Acculturative Stress					39.99	13.74	15–75
2. Anxiety	0.170^*^				4.64	5.09	0–21
3. Somatization	0.176^*^	0.661^**^			5.67	5.48	0–30
4. Resilience	−0.289^**^	−0.315^**^	−0.361^***^		19.98	4.14	7–30

^*^ *p* < .05.

^**^ *p* < .01.

^***^ *p* < .001.

### 3.2 Primary analyses

#### 3.2.1 Mediations

**Figure 2:**
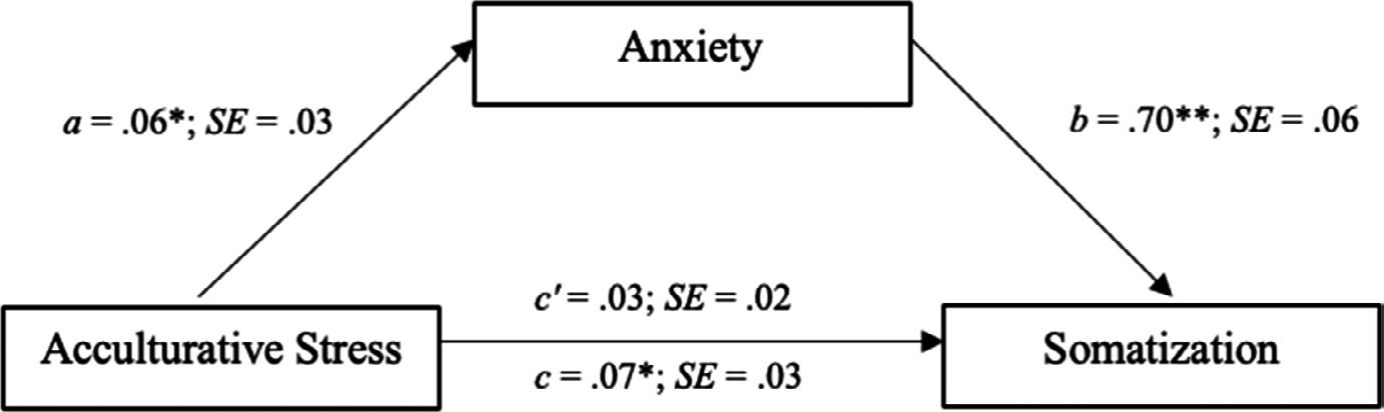
Statistical representation of anxiety as a mediator of the relationship between acculturative stress and somatization

In the mediation model (Figure 2), acculturative stress was specified to have a direct effect on somatization, as well as an indirect effect through anxiety, using 5,000 bootstrap samples. The direct paths from acculturative stress to anxiety (*b* = 0.06, *p* = .015) and from anxiety to somatization (*b* = 0.70, *p* < .001) were both statistically significant. Further, the indirect effect of acculturative stress on somatization through anxiety was statistically significant (*b* = 0.04, 95% CI [0.005, 0.08]), indicating a full mediation because the direct path from acculturative stress to somatization (c′ path) was not statically significant in the model (*b* = 0.026, *p* = .217).

#### 3.2.2 Moderated mediation

**Figure 3:**
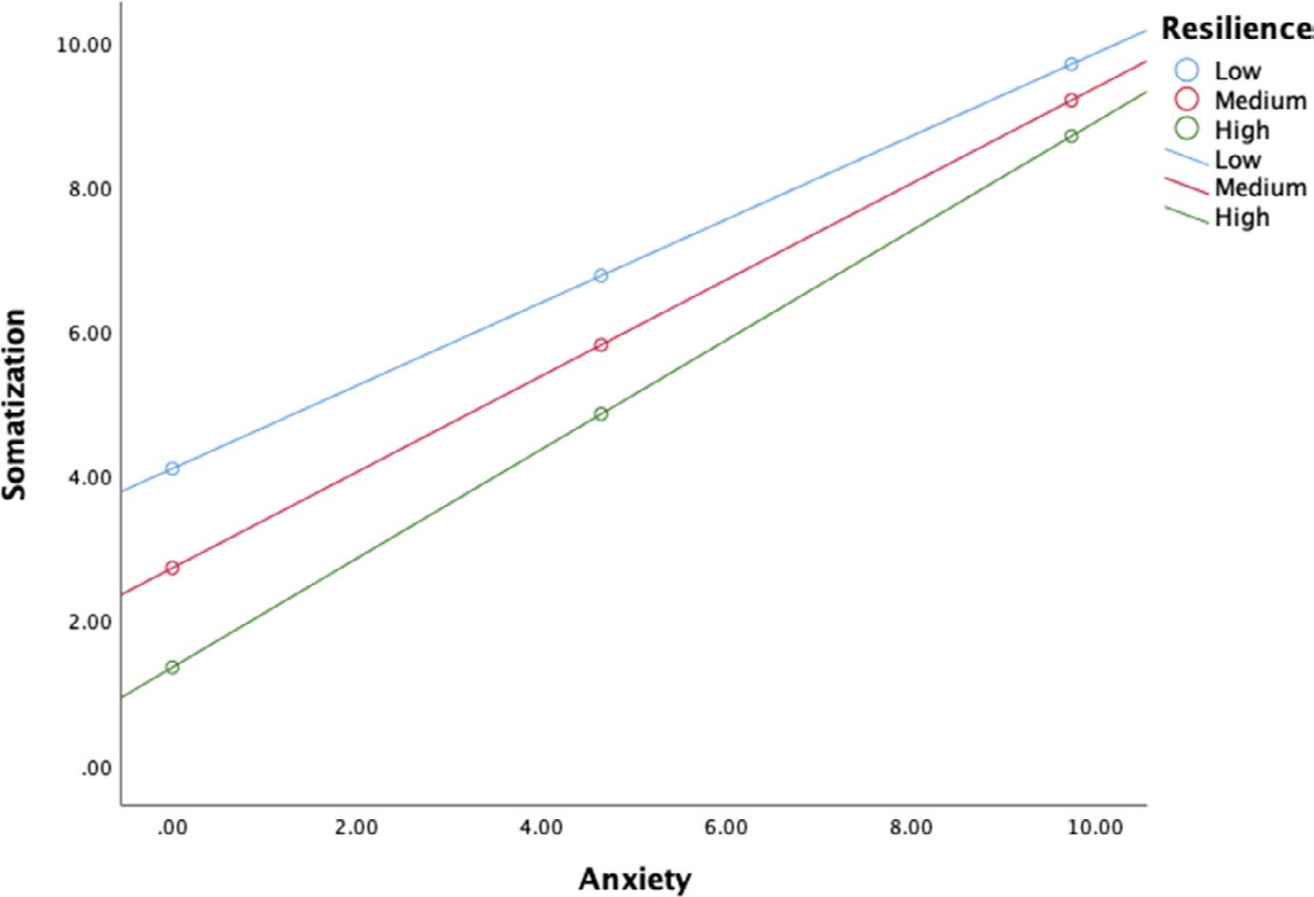
Resilience moderating the relation between anxiety and somatization

**Figure 4:**
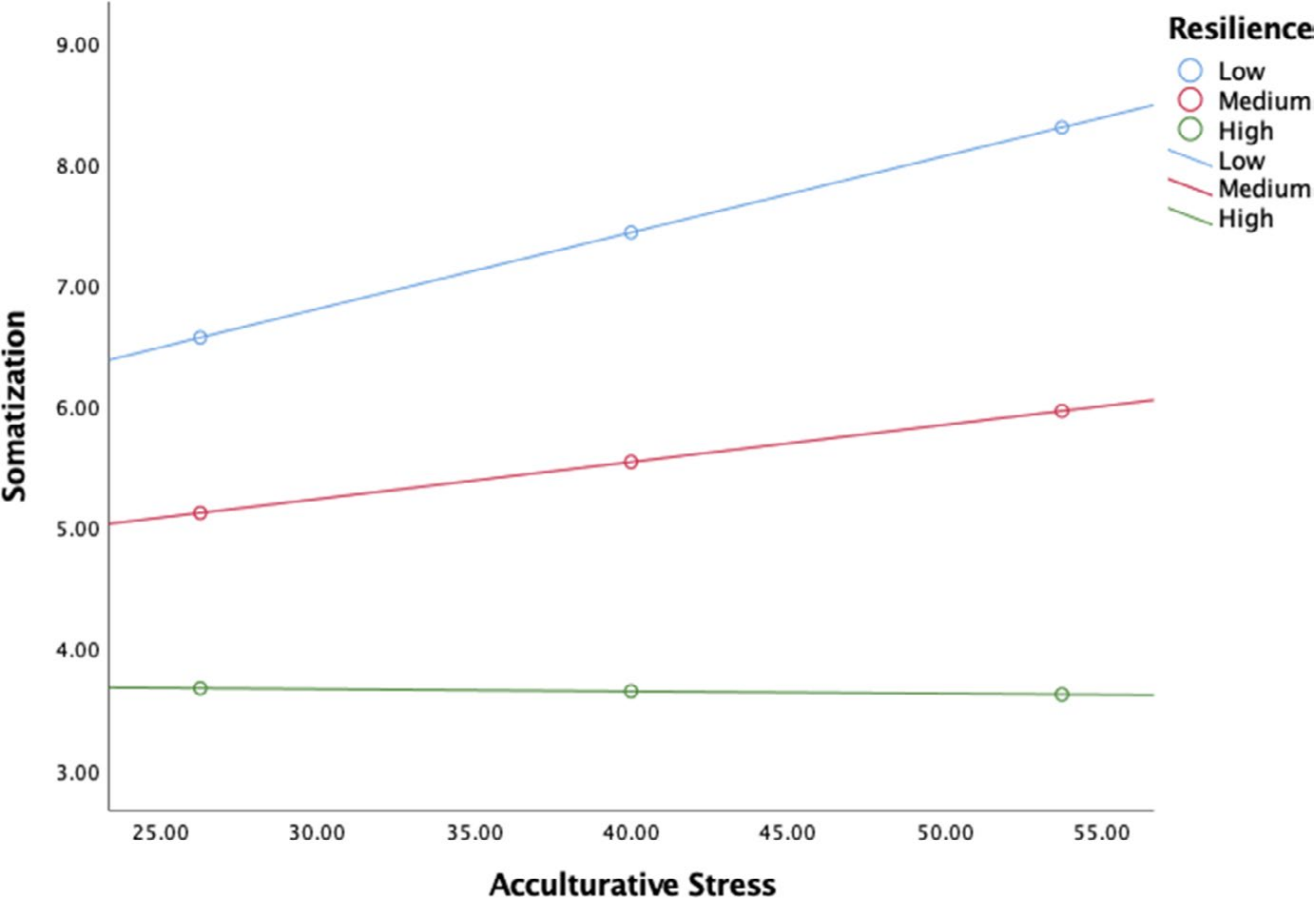
Resilience moderating the relation between acculturative stress and somatization

In order to determine whether the mediational effect from acculturative stress through anxiety to somatization differed as a function of participants’ level of resilience (i.e., moderated mediation), a conditional process model was conducted. The overall model investigating somatization was significant, *F*(5, 198) = 36.99, *p* < .001, *R* ^2^ = 0.48. There was not a significant direct effect of acculturative stress to anxiety (a path) when resilience was included in the model (*b* = 0.03, *p* = .222). Resilience was negatively associated with anxiety (*b* = −0.36, *p* < .001). The acculturative stress × resilience interaction with anxiety as the criterion variable was not significant (*b* = −0.001, *p* = .861). Additionally, the direct effect of anxiety (b path) was positively associated with somatization (*b* = 0.66, *p* < .001) when resilience was included in the model. Acculturative stress was not significant (c path) when resilience was included in the model (*b* = 0.01, *p* = .732). The interaction between anxiety × resilience was significant (Figure 3; *b* = 0.03, *p* = .021) as well as the interaction between acculturative stress x resilience (Figure 4; *b* = −0.01, *p* = .041), suggesting that resilience buffered both of these effects on somatization. Despite these moderations, there were no conditional indirect effects of acculturative stress on somatization based on the level of resilience.

## 4 DISCUSSION

The purpose of this study was to explore relations among acculturative stress, anxiety, somatization, and resilience in a sample of Latinx immigrants living in the United States. Acculturative stress was positively related to both anxiety and somatization, and the relation between acculturative stress and somatization occurred through anxiety. Resilience buffered the relation between acculturative stress and somatization, though very slighly exacerbated the relation between anxiety and somatization. The current buffering finding parallels Meyer's (2003) minority stress model as acculturative stress, a minority stressor, negatively impacted Latinx immigrants’ mental and physical health expressed in anxiety and somatization. Similarly, the findings also support that protective cultural factors expressed in resilience can weaken the impact of these daily life stressors experienced by Latinx immigrants.

In the current study, 34.9% of Latinx immigrants reported clinically significant levels of anxiety. This rate was a bit higher than previously found among Latinxs living in the United States and suggests a need for mental health screenings and services for this population (Schraufnagel et al., 2006). As mental health concerns, including anxiety, are commonly and often first disclosed to primary care physicians (Goldberg, 2011; Goldberg et al., 2012), the current findings support the American Medical Associations’ recommendation to primary care providers to screen patients for mental health disorders (Talen, 2013). The GAD‐2, a two‐item measure for anxiety, has been found to be an excellent screening tool in primary care to identify patients with common anxiety disorders (Kroenke et al., 2007). As comorbidity between anxiety and somatization has been found in this study as well as other Latinx samples (Escobar et al., 2010), healthcare providers should consider clinically investigating possible biopsychosocial influences on anxiety disorders in Latinx immigrants and refer patients to mental health providers engaging in culturally sensitive empirically based treatment.

Acculturative stress was positively associated with anxiety and somatization, but inversely associated with resilience in the current sample. These findings were congruent with previous research investigating the connection between mental and physical health in Latinx immigrants (Bauer et al., 2012), although the positive relationship between acculturative stress and somatization is the first known occurrence in the research literature to the authors’ knowledge. This direct relationship is congruent with Meyer's (2003) minority stress model as acculturative stress (a minority stressor) likely impacted the somatization of Latinx immigrants through a somatic presentation. As anxiety fully mediated this relationship, suggesting a possible pathway by which this relationship operates, acculturative stress may increase Latinx immigrants’ anxiety, which results in increased rates of somatic complaints. This finding is congruent with Kroenke et al.'s (1997) study tying somatic symptoms to a twofold increased risk of an anxiety disorder.

Resilience was found to be negatively correlated with acculturative stress, anxiety, and somatization which is not surprising due to the high levels of cultural strengths found within Latin American cultures (Luthar et al., 2000). Resilience buffered the relationship between accululturative stress and somatization (Figure 4). Latinx resilience is grounded in cultural strengths including familism, religiosity, biculturalism, personalism, and community support (Luthar et al., 2000), which may have played a role. As a result, Latinx immigrants might benefit from engagement in cultural activities, contributing to resilience and buffering them against both anxiety and somatization. Research in Latinx mental health also previously documented the protective function of cultural strengths on the negative effects of acculturative stress (Finch & Vega, 2003; Wong et al., 2017). Resilience was also found to slightly exacerbate the relations between somatization and anxiety (Figure 3) which may be due, conversely, to multicollinearity in the regression rather than a true effect.

The results of this study provide support for the use of integrated care services in medical settings to address the possible biopsychosocial–cultural presentation of somatization in Latinx immigrants in the United States. Specifically, the results contribute to the understanding of the relations among acculturative stress, anxiety, somatization, and resilience in Latinx immigrants. Both physicians and mental health providers working with Latinx immigrants are advised to assess for the level of acculturative stress in Latinx immigrant patients and its potential to contribute to the presentation of anxiety and additional medical concerns. When creating treatment plans and compiling possible recommendations for somatic complaints, healthcare providers are encouraged to engage in integrated health care by referring Latinx immigrant patients to social work and behavioral health services. Behavioral health clinicians targeting the impact of acculturative stress are recommended to assess the level of anxiety and somatization in Latinx immigrants to aid in treatment planning and implementation. The utilization of integrated health services may reduce acculturative stress in Latinx immigrants, possibly improving anxiety and somatic symptoms. As resilience was found to be a buffer, physicians, behavioral health clinicians, and social workers are encouraged to support and integrate Latinx cultural strengths of familism, social networks, and religious practices into treatment. Integrated health services targeting behavioral health concerns and integrating Latinx cultural strengths may reduce the impact of acculturative stress on the mental and physical health of Latinx patients.

### 4.1 Limitations

The current study's findings are to be considered in the context of several limitations that present opportunities for future research. As there are multiple subethnicities within the larger Latinx population, these results are not fully generalizable to all Latinx immigrants living in the United States, especially for those whose country of origin is other Central and South American countries. Any further research and clinical applications that are recommended are best tailored to the specific values and beliefs of that particular culture (i.e., Mexican, Cuban, Bolivian, Argentinian). The current study did not assess for differences according to gender, acculturation levels, socioeconomic statuses, nor length of stay in the United States. Another limitation is the lack of investigation of differing types of somatization as Latinx immigrants may be experiencing higher levels of a specific type of somatic symptom. In addition, the current study only investigated one type of minority stressor, acculturative stress, when Latinx immigrants also experience daily discrimination which has been found to impact Latinx mental and physical health (Flores et al., 2008). An inclusion criterion for the study was the ability to read and write in Spanish, and due to lower literacy levels in Latinxs in the United States, this criterion excluded a subset of Latinx immigrants. As one‐fifth of Latinx immigrants living in the United States are illiterate, this study may have excluded participants experiencing greater minority stressors due to systemic barriers (Taylor et al., 2012).

## 5 CONCLUSION

As somatic symptoms are the leading cause of outpatient medical visits and are one of the leading causes of under‐recognition and undertreatment of mental health concerns, investigation of factors that contribute to somatization in Latinx immigrants is necessary. The current study adds to the understanding of the relations among acculturative stress, anxiety, and somatization in Latinx immigrants living in the United States. The current investigation is the first study that investigated both the direct effect of acculturative stress on somatization and the mediational effect of anxiety. Additionally, the present investigation was also the first to examine the impact of resilience as a potential moderating effect among the relations among acculturative stress, anxiety, and somatization. Clinicians and healthcare providers are advised to assess for the impact of acculturative stress and anxiety when discussing treatment plans of somatization with Latinx immigrants living in the United States and engage in integrated health care by referring Latinx immigrant patients to behavioral health services as needed. Since resilience was found to weaken the relationships between acculturative stress and somatization, it is recommended that future interventions targeting Latinx immigrant somatization incorporate Latinx cultural strengths that drive resilience.

## CONFLICT OF INTEREST

The authors have no competing interests to report.

## AUTHOR CONTRIBUTIONS

AMP, ANC, and PBP conceptualized the project. ANC and AMP collected the data. ANC and PBP analyzed the data. ANC, PBP, and AMP wrote the manuscript. PBP edited the manuscript.

### Peer Review

The peer review history for this article is available at https://publons.com/publon/10.1002/brb3.1863.

## Data Availability

Data are available by request to the corresponding author.
